# Berberine and Palmatine Distribution Across Plant Organs in *Berberis darwinii*: Basis for Selecting Superior-Producing Accessions

**DOI:** 10.3390/molecules30081849

**Published:** 2025-04-20

**Authors:** Manuel Chacón-Fuentes, César Burgos-Díaz, Mauricio Opazo-Navarrete, Alan Mercado, Fernando Westermeyer

**Affiliations:** Agriaquaculture Nutritional Genomic Center, CGNA, Temuco 4780000, Chile; cesar.burgos@cgna.cl (C.B.-D.); mauricio.opazo@cgna.cl (M.O.-N.); alan.mercado@cgna.cl (A.M.); fernando.westermeyer@cgna.cl (F.W.)

**Keywords:** *Berberis darwinii*, alkaloids, berberine, palmatine, regional variation, tissue-specific differences, plant selection

## Abstract

*Berberis darwinii*, known for its bioactive alkaloids like berberine and palmatine, has gained attention for its medicinal properties. However, comprehensive studies on the specific bioactive molecules of Michay are lacking, as previous research has primarily focused on wild plants. Therefore, this study proposes to evaluate the alkaloid content in various tissues of *B. darwinii* collected from different locations, aiming to identify high-yielding accessions suitable for consistent bioactive alkaloid production. This research focuses on plants from a cultivated Michay orchard established five years ago. Leaves, stems, roots, and fruits from 96 accessions of Michay were collected to obtain an alkaloidal extract used for the characterization and comprehensive analysis of bioactive alkaloids through high-performance liquid chromatography. Based on these results, a search for the main outliers was conducted to identify the accessions with the highest alkaloid production. The results showed that roots had the highest concentrations of both berberine and palmatine, followed by stems, while leaves and seeds had lower levels, and the pulp from fruits had no detectable alkaloids. Notably, alkaloid concentrations reached up to 30,806 µg/g in roots, with accession C2P18 standing out for its combined total of 20,827.74 µg/g of berberine and 9978.27 µg/g of palmatine. Accession C3P26 showed the highest berberine concentration at 26,482.20 µg/g. These values underscore the wide variation in alkaloid accumulation and highlight the potential for selecting elite accessions with exceptionally high yields. These findings highlight the importance of plant selection for optimal alkaloid extraction. Choosing high-yielding accessions and standardizing cultivation practices will ensure a stable supply of berberine and palmatine for pharmaceutical, nutraceutical, and food industry applications.

## 1. Introduction

Michay (*Berberis darwinii*) is a native shrub from the temperate forests of southern South America [1,2], primarily distributed in southern Chile, from the Ñuble to Aysén regions, as well as in the mountainous areas of Argentine Patagonia [3,4]. This species typically grows between 1 and 3 m tall. Its leaves are abundant, arranged in groups of 3 to 5 at the base of strong thorns, and have a leathery texture. The plant produces raceme inflorescences, with fruits featuring a style measuring 1–3 mm in length. Its flowers cluster together, displaying six orange sepals and a bottle-shaped pistil. The fruit is a small, round berry that ripens from green to black and contains 3 to 4 seeds [5]. Michay berries have long been used in culinary applications, serving as decoration in pastries and as ingredients in juices, sweets, and syrups [6,7]. Additionally, the fruits, leaves, stems, and roots of *B. darwinii* have been traditionally used in medicine to treat conditions such as fever, inflammation, stomachaches, and infections, owing to their bioactive compound content [8]. The plant is particularly known for alleviating stomach pain, indigestion, and colitis due to its high antioxidant content [7]. Hence, this shrub is highly valued for its dual role in medicine and gastronomy, largely attributed to its significant antioxidant properties. *Berberis darwinii* is rich in various phytochemical molecules found in its stems, fruits, leaves, and roots [9]. Notably, it contains several bioactive alkaloids and polyphenols, which are considered particularly significant due to their potential bioactive properties [10,11]. Currently, Michay has been studied for its benzylisoquinoline-type alkaloids, particularly berberine and palmatine. However, others have also been reported, such as magallanesine, dihydrorugosinone, rugosinone, nuevamine, santiagonamine, and chiloenamine [12,13,14,15]. Additionally, Perez-San Martin et al. [16] reported that the main alkaloids identified in *B. darwinii* include berberine, palmatine, jatrorrhizine, columbamine, laurifoline, magnoflorine, tetrahydropalmatine, and thalifendine. The significance of this group of compounds, particularly berberine and palmatine, lies in their reported health benefits [17,18,19,20,21]. This well-known phytochemical is primarily extracted from the roots of various plant species, including *Berberis vulgaris*, *B. aristata*, *B. aquifolium*, *Hydrastis canadensis*, *Phellodendron chinense*, and *Coptis rhizomes* [21]. Berberine is a yellow crystalline isoquinoline alkaloid with a long history of use in traditional Chinese and Ayurvedic medicine [22]. Recent research highlights its broad therapeutic potential, demonstrating effectiveness against multiple conditions such as diabetes, hypertension, depression, obesity, inflammation, and cancer [23,24,25,26]. Moreover, berberine sulfate and hydrochloride have been identified as efficient herbal treatments [27]. For example, studies suggest that berberine holds promise as a potential drug candidate, particularly in cancer therapy [28] and the treatment of diseases such as diabetes and Alzheimer’s. However, due to its hydrophilic nature, berberine exhibits low oral bioavailability. In addition to berberine, another structurally related protoberberine alkaloid of interest is palmatine, which shares similar pharmacological properties and is commonly found in *Rhizoma coptidis*, *Phellodendron*, and various other Chinese medicinal plants [29]. Like berberine, palmatine has been studied for its diverse therapeutic potential, making its stable production in cultivated Michay plants equally important. Similar to berberine, it has attracted scientific interest due to its wide range of pharmacological properties, including anticancer, neuroprotective, antiviral, antibacterial, anti-inflammatory, antioxidant, and lipid-regulating effects [30]. In the context of fibrotic diseases, palmatine has shown remarkable anti-fibrotic activity, particularly in liver fibrosis. Its mechanisms of action involve alleviating metabolic disorders and restoring gut microbiota balance, contributing to overall liver health [31,32,33]. Given the medicinal relevance of these alkaloids, establishing consistent concentrations in cultivated Michay plants could enhance their potential applications.

To date, all existing studies on these bioactive alkaloids have focused exclusively on wild populations of Michay [12,13,14,15,16]. This is particularly relevant because there is no dedicated cultivation area for this plant at either the national or global level, further complicating its domestication and the stable production of these compounds. Moreover, in other native berry species, domestication has been shown to induce metabolic changes [34]. Given this, the transition from wild populations to cultivated systems could significantly influence alkaloid biosynthesis. Consequently, the production of berberine and palmatine will not only depend on environmental factors but also on the selected genotype, introducing inherent variability in alkaloid concentrations and posing a challenge to achieving consistent, standardized yields [18]. Therefore, the variability in compound concentrations among different plants is substantial, making it difficult to establish precise measurements of these alkaloids across a given region. Thus, the inherent variability in alkaloid concentrations among different *B. darwinii* plants poses significant challenges for their medicinal and commercial utilization. This variability is driven by a complex interplay of genetic, environmental, developmental, and physiological factors that influence the biosynthesis, accumulation, and distribution of berberine and palmatine [19,20]. Understanding these factors is essential for optimizing plant selection, improving cultivation strategies, and achieving a more consistent production of these valuable bioactive compounds.

Based on the above considerations, the primary objective of this study is to characterize the content of bioactive alkaloids, specifically berberine and palmatine, in different tissues (leaves, stems, roots, seeds, and fruit pulp) of a population of 96 accessions of Michay to achieve the selection of a superior accession. Unlike previous research, which has predominantly focused on wild populations, this study is based on plants from a cultivated Michay orchard established five years ago. By identifying high-yielding accessions suitable for consistent and sustainable alkaloid production, this research aims to support the domestication and propagation of superior accessions, ensuring a more stable and standardized supply of these bioactive compounds. Therefore, this study represents the first step in selecting the most productive Michay plants in terms of bioactive alkaloid molecule production. The future perspective of this work is to begin the propagation of these plants to establish the first germplasm bank of Michay, with selected clones that surpass the evaluated populations. These efforts mark the beginning of an exciting new chapter in the sustainable cultivation of Michay, where future generations of these plants could provide an invaluable resource for the pharmaceutical industry. As we advance, the potential to produce high-quality bioactive compounds from these superior accessions may reshape how we source and utilize natural remedies for a variety of therapeutic applications.

## 2. Results

The variability in alkaloid molecules concentration among *B. darwinii* accessions from different locations provided key information for selecting high-yielding individuals in bioactive compounds (berberine and palmatine). The following section presents the results of the quantification of berberine and palmatine in samples collected from five locations in Chile, allowing the identification of significant differences in the accumulation of these alkaloids. An additional noteworthy aspect of the spatial alkaloid analysis in Michay was that, upon separating the fruit into pulp and seed, the pulp from all accessions failed to exhibit detectable levels of either berberine or palmatine. Consequently, for the analysis and reporting of the results, the pulp was excluded.

### 2.1. Regional Variability in Alkaloid Content of B. darwinii: Distinct Patterns of Berberine and Palmatine Accumulation Across Locations

**Figure 1:**
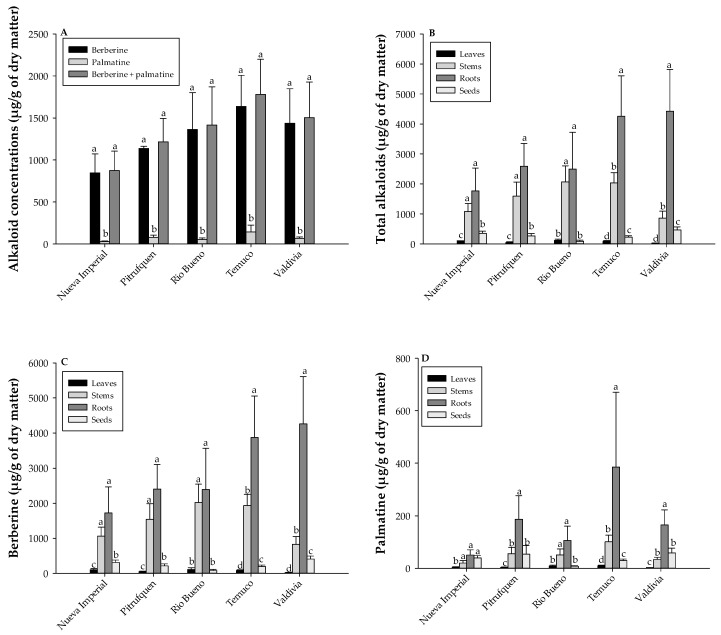
Berberine, palmatine, and berberine + palmatine concentrations (µg/g of DM) in different locations (**A**). Total alkaloid content (berberine + palmatine) in different organs of the Michay plant (**B**). Berberine content in different organs of the Michay plant (**C**). Palmatine content in different organs of the Michay plant (**D**). Different letters indicate significant differences (*p* < 0.05) among treatments within each location. Error bars represent the standard error (SE).

Figure 1A presents the alkaloid concentrations of berberine and palmatine across different locations, highlighting significant differences. The total amounts from all tested plant organs were analyzed. In Nueva Imperial, berberine concentration was 846.39 ± 225.63 µg/g, significantly higher than palmatine at 28.27 ± 6.48 µg/g, with a total of 874.65 ± 231.03 µg/g. In Pitrufquén, berberine reached 1136.34 ± 27.45 µg/g, while palmatine was 78.09 ± 27.45 µg/g, summing to 1214.43 ± 279.97 µg/g. Río Bueno showed a berberine concentration of 1364.59 ± 436.67 µg/g, significantly higher than palmatine at 52.15 ± 19.90 µg/g, with a total of 1416.74 ± 454.41 µg/g. In Temuco, berberine was 1636.77 ± 370.87 µg/g, significantly higher than palmatine at 143.48 ± 81.48 µg/g, resulting in a total of 1780.25 ± 418.68 µg/g. Finally, in Valdivia, berberine concentration was 1437.31 ± 410.09 µg/g, while palmatine was significantly lower at 65.94 ± 17.23 µg/g, with a total of 1503.25 ± 424.22 µg/g. Although some groups exhibited mild deviations from homogeneity, Tukey’s test was chosen as the post hoc method because it is robust and widely used when comparing all possible pairwise group differences, especially when group sizes are equal or nearly equal, as in our design.

### 2.2. Tissue-Specific Distribution of Alkaloids in B. darwinii: Roots as the Primary Site of Berberine Accumulation, While Stems Concentrate Palmatine

In Nueva Imperial, roots exhibited the highest total alkaloid concentration (1771.96 ± 760.81 µg/g), significantly surpassing stems (1078.88 ± 267.68 µg/g), leaves (107.67 ± 1.71 µg/g), and seeds (352.41 ± 72.45 µg/g). In Pitrufquén, roots also accumulated the highest alkaloid content (2590.96 ± 757.28 µg/g), followed by stems (1596.31 ± 467.01 µg/g), leaves (48.28 ± 21.14 µg/g), and seeds (268.67 ± 78.55 µg/g). Río Bueno presented a similar pattern, with roots containing the highest concentration (2495.40 ± 1226.94 µg/g), followed by stems (2071.31 ± 531.43 µg/g), leaves (118.68 ± 42.94 µg/g), and seeds (89.38 ± 35.08 µg/g). In Temuco, roots displayed the highest alkaloid levels (4256.90 ± 1346.59 µg/g), significantly exceeding stems (2035.56 ± 339.59 µg/g), leaves (92.51 ± 20.38 µg/g), and seeds (223.87 ± 47.74 µg/g). Finally, in Valdivia, roots again accumulated the highest alkaloid content (4427.23 ± 1392.61 µg/g), significantly higher than stems (862.23 ± 232.11 µg/g), leaves (23.77 ± 7.05 µg/g), and seeds (467.72 ± 101.53 µg/g) (Figure 1B).

When analyzing the berberine content separately from the total alkaloid content shown in Figure 1C, it became evident that in Nueva Imperial, roots exhibited the highest berberine concentration (1721.19 ± 743.28 µg/g), followed by stems (1058.98 ± 259.95 µg/g), seeds (313.03 ± 66.61 µg/g), and leaves (102.88 ± 32.88 µg/g). Pitrufquén showed a similar pattern, with roots accumulating the highest levels (2404.02 ± 696.11 µg/g), followed by stems (1540.94 ± 448.72 µg/g), seeds (214.48 ± 55.18 µg/g), and leaves (44.55 ± 20.17 µg/g). In Río Bueno, berberine concentration was also highest in roots (2389.51 ± 1174.42 µg/g), followed by stems (2020.15 ± 524.81 µg/g), seeds (81.54 ± 33.50 µg/g), and leaves (109.50 ± 51.17 µg/g). In Temuco, roots exhibited the highest berberine content (3870.99 ± 1182.44 µg/g), significantly higher than in stems (1934.61 ± 321.67 µg/g), seeds (194.11 ± 43.72 µg/g), and leaves (82.53 ± 19.20 µg/g). Lastly, in Valdivia, roots accumulated the highest berberine concentration (4262.19 ± 1347.01 µg/g), followed by stems (828.20 ± 225.00 µg/g), seeds (409.03 ± 85.87 µg/g), and leaves (20.88 ± 6.43 µg/g).

Significant variations in palmatine content among localities were observed when analyzing different plant organs (Figure 1D). In Nueva Imperial, stems exhibited the highest palmatine concentration (50.77 ± 19.59 µg/g), followed by roots (39.39 ± 9.08 µg/g), seeds (19.90 ± 9.48 µg/g), and leaves (4.79 ± 1.71 µg/g). In Pitrufquén, stems also accumulated the highest levels (186.93 ± 89.81 µg/g), significantly higher than in roots (54.19 ± 32.14 µg/g), seeds (55.38 ± 24.50 µg/g), and leaves (3.73 ± 1.39 µg/g). Río Bueno showed a similar pattern, with stems presenting the highest palmatine content (105.89 ± 55.02 µg/g), followed by roots (7.84 ± 2.26 µg/g), seeds (51.16 ± 22.76 µg/g), and leaves (9.17 ± 3.27 µg/g). In Temuco, stems exhibited the highest palmatine concentration (385.91 ± 284.08 µg/g), significantly surpassing roots (29.76 ± 5.85 µg/g), seeds (100.95 ± 25.05 µg/g), and leaves (9.98 ± 2.48 µg/g). Lastly, in Valdivia, stems again showed the highest palmatine levels (165.04 ± 57.69 µg/g), followed by roots (58.69 ± 18.06 µg/g), seeds (34.03 ± 8.14 µg/g), and leaves (2.89 ± 0.71 µg/g). These results highlighted that stems generally accumulated the highest palmatine content across all studied localities, with particularly elevated levels in Temuco and Pitrufquén.

### 2.3. Variability of Alkaloid Content Across Plant Organs and Locations

The coefficient of variation (CV) was used to assess the variability of berberine and palmatine concentrations across different plant organs and collection zones (Table 1). In leaf tissues, CV values were consistently high for both alkaloids, exceeding 98% in all locations. The highest CV for berberine was observed in Pitrufquén (163.23%), while palmatine reached its maximum variability in Nueva Imperial (147.07%). These values suggest significant heterogeneity in alkaloid accumulation within leaf samples, possibly due to environmental or genetic factors. In stem tissues, berberine showed moderate to high variability, with CV values ranging from 86.16% (Rio Bueno) to 115.26% (Valdivia). Palmatine presented greater variation across all locations, with CV values above 100%, peaking at 202.10% in Nueva Imperial. This indicates substantial fluctuation in palmatine levels among individual stems, even within the same population. Root samples exhibited the highest overall CVs, especially for palmatine. Berberine variability reached up to 183.90% (Rio Bueno), while palmatine showed an extreme CV of 435.50% in Temuco. These results suggest marked differences in alkaloid storage or biosynthetic activity among root systems of different individuals. In seeds, the CV values were comparatively lower than in other organs. Berberine variability ranged from 76.72% (Nueva Imperial) to 117.04% (Temuco), and palmatine from 81.69% (Rio Bueno) to 187.57% (Pitrufquén). These results suggest a more stable pattern of alkaloid accumulation during seed development, possibly reflecting tighter physiological regulation or lower environmental sensitivity.

### 2.4. Outliers Identification

The high standard error value indicates a great phenotypic plasticity within the accessions in each locality. This suggests the probable presence of accessions with high berberine and palmatine production. Therefore, identifying these outliers is necessary to pinpoint potential superior accessions.

**Figure 2:**
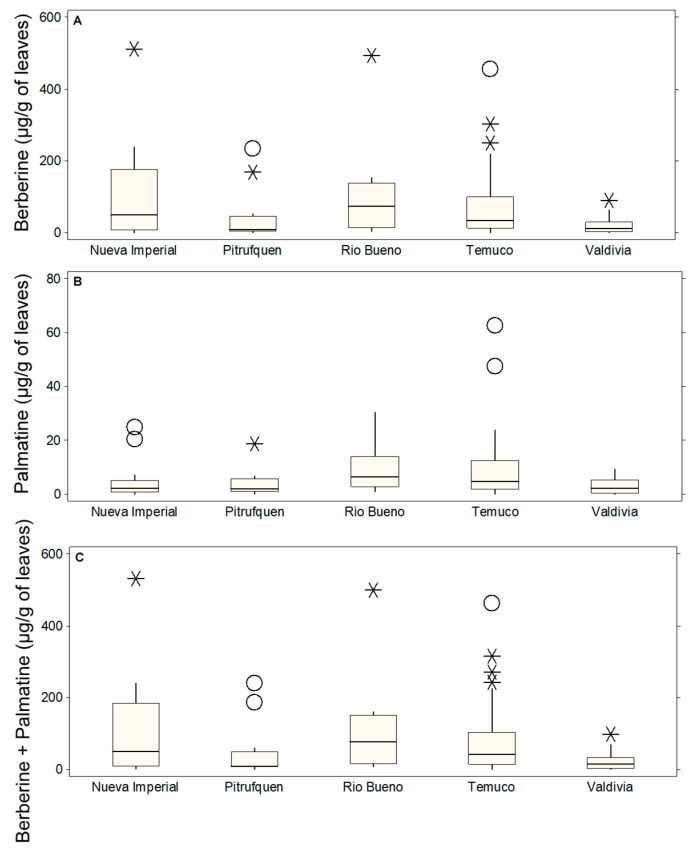
Box plots showing the concentrations of leaves alkaloids (µg/g of DM) in different locations: Nueva Imperial, Pitrufquén, Río Bueno, Temuco, and Valdivia. (**A**) Berberine, (**B**) palmatine, and (**C**) berberine + palmatine. The boxes represent the interquartile range (IQR), the horizontal line within each box indicates the median, whiskers extend to 1.5 times the IQR, and circles denote probable outliers and asterisks denote possible outliers.

Figure 2 illustrates the distribution of alkaloid content in Michay leaves across different localities: Nueva Imperial, Pitrufquén, Río Bueno, Temuco, and Valdivia. Berberine content (Figure 2A) exhibited the highest variability in Nueva Imperial and Temuco, with extreme outliers reaching up to 511.02 µg/g in Nueva Imperial (C1P5, *) and 455.63 µg/g in Temuco (C3P22, O), while Río Bueno also displayed a high value of 493.10 µg/g (C4P6, *). In Temuco, additional values included 303.49 µg/g (C1P19, *) and 249.03 µg/g (C2P18, *). In contrast, Pitrufquén presented lower median concentrations, with individual accessions reaching 233.82 µg/g (C1P3, O) and 167.81 µg/g (C1P2, *), whereas Valdivia had the lowest values, with its highest outlier at 89.36 µg/g (C1P10, *). Palmatine content (Figure 2B) was significantly lower than that of berberine, with a maximum recorded value of 62.57 µg/g in Temuco (C4P19, O) and 47.41 µg/g in another Temuco accession (C2P24, O). Nueva Imperial also presented slightly elevated values, with a maximum of 24.78 µg/g (C1P6, O), while Río Bueno and Pitrufquén remained below 20 µg/g, with outliers reaching 20.31 µg/g (C1P5, O) and 18.71 µg/g (C1P2, *), respectively.

Valdivia showed consistently low palmatine levels, with no significant outliers. The combined berberine + palmatine content (Figure 2C) followed a distribution similar to berberine, given its dominance, with the highest values observed in Nueva Imperial at 531.33 µg/g (C1P5, *), Río Bueno at 499.73 µg/g (C4P6, *), and Temuco at 462.75 µg/g (C3P22, O). Other Temuco accessions also displayed considerable variation, with values such as 314.93 µg/g (C1P19, *), 270.50 µg/g (C2P18, *), and 242.96 µg/g (C2P23, *). In contrast, Pitrufquén exhibited more moderate values, with peaks at 240.11 µg/g (C1P3, O) and 186.52 µg/g (C1P2, O), while Valdivia remained the lowest, with its highest recorded value at only 98.48 µg/g (C1P10, *). The presence of numerous extreme values in Nueva Imperial, Temuco, and Río Bueno, particularly in berberine content, suggested that environmental or genetic factors might have influenced alkaloid accumulation. Pitrufquén and Valdivia, despite some minor outliers, consistently exhibited the lowest median values, indicating that these locations might have been less favorable for alkaloid production in Michay leaves.

**Figure 3:**
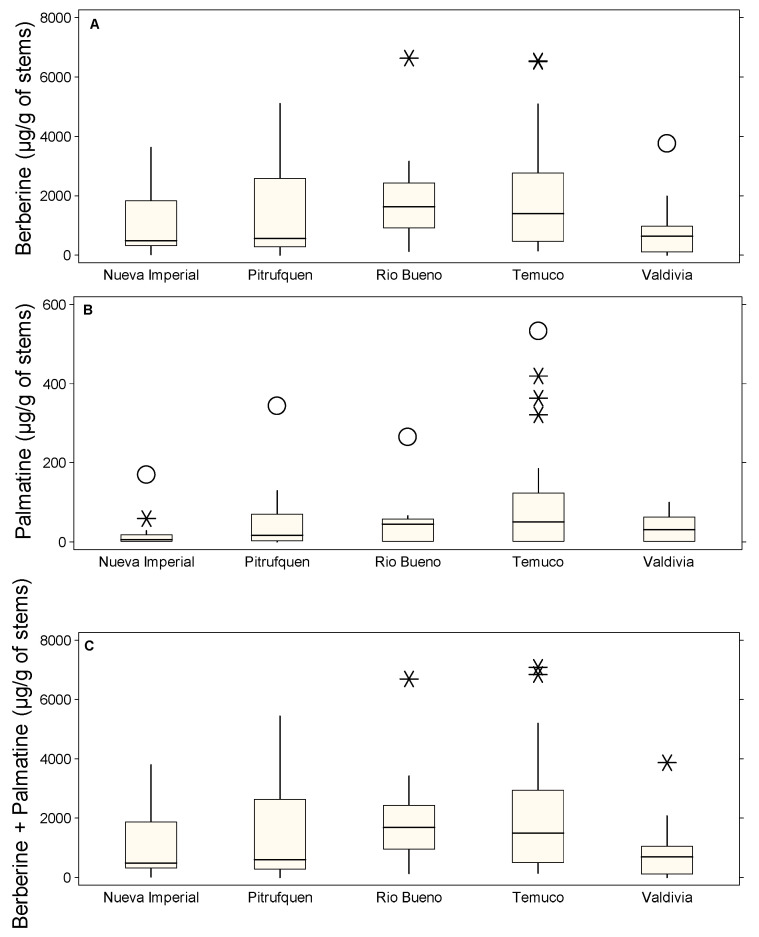
Box plots showing the concentrations of stems alkaloids (µg/g of DM) in different locations: Nueva Imperial, Pitrufquén, Río Bueno, Temuco, and Valdivia. (**A**) Berberine, (**B**) palmatine, and (**C**) berberine + palmatine. The boxes represent the interquartile range (IQR), the horizontal line within each box indicates the median, whiskers extend to 1.5 times the IQR, and circles denote probable outliers and asterisks denote possible outliers.

Figure 3 illustrates the concentrations of berberine (Figure 3A), palmatine (Figure 3B), and their sum (Figure 3C) extracted from Michay stems across different locations, highlighting the presence of multiple outliers. In Figure 3A, Temuco exhibits the highest variability, with extreme values such as 6544.23 µg/g (C1P25, *), indicating significant fluctuations in berberine accumulation. Río Bueno also showed a high outlier, reaching 6639.58 µg/g (C2P1, *). Valdivia presented a moderate level of variability, with a maximum recorded value of 3766.27 µg/g (C3P8, O). In contrast, Nueva Imperial and Pitrufquén had no reported berberine outlier values. Figure 3B revealed considerably lower palmatine concentrations across all locations. Temuco displayed the widest spread, with multiple outliers such as 533.41 µg/g (C1P25, O), 418.75 µg/g (C2P24, *), 362.62 µg/g (C2P26, *), and 321.45 µg/g (C1P26, *). Río Bueno showed a relatively lower concentration, with a peak at 265.32 µg/g (C2P13, O). Pitrufquén exhibited a moderate level at 342.78 µg/g (C1P13, O), while Nueva Imperial presented a maximum value of 170.03 µg/g (C1P5, O) and 58.53 µg/g (C1P6, *), the lowest among all locations. In Figure 3C (berberine + palmatine), the trend closely mirrored that of berberine, as it was the dominant compound. Temuco presented the highest combined concentrations, with values such as 7077.64 µg/g (C1P25, *) and 6842.67 µg/g (C1P26, *). Río Bueno followed with an extreme outlier of 6684.27 µg/g (C2P1, *). Valdivia showed a lower but still notable concentration of 3865.38 µg/g (C3P8, *).

**Figure 4:**
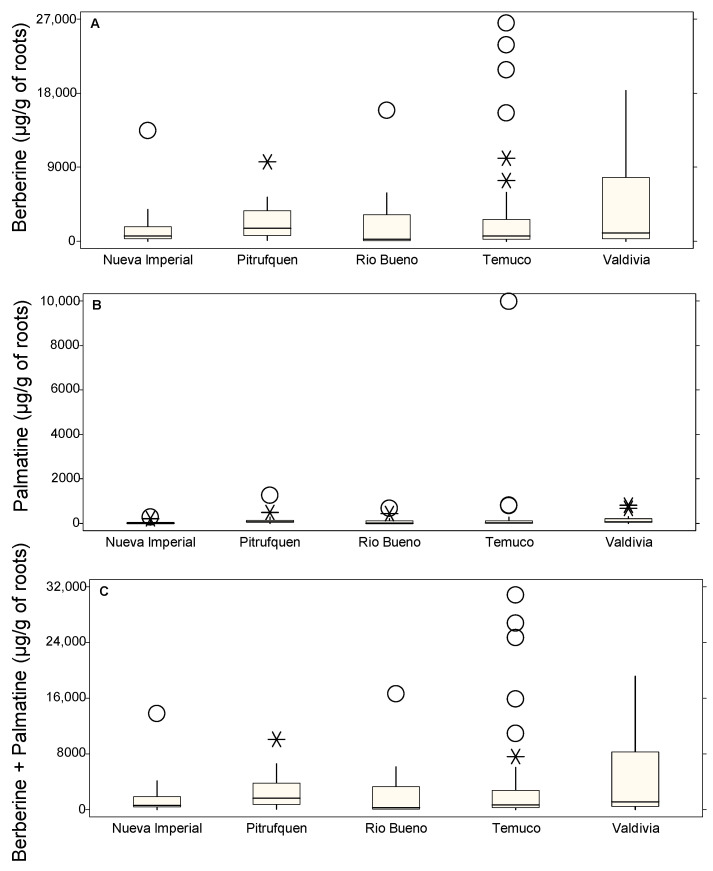
Box plots showing the concentrations of roots alkaloids (µg/g of DM) in different locations: Nueva Imperial, Pitrufquén, Río Bueno, Temuco, and Valdivia. (**A**) Berberine, (**B**) palmatine, and (**C**) berberine + palmatine. The boxes represent the interquartile range (IQR), the horizontal line within each box indicates the median, whiskers extend to 1.5 times the IQR, and circles denote probable outliers and asterisks denote possible outliers.

Figure 4 illustrates the distribution of alkaloid content in Michay roots across different localities: Nueva Imperial, Pitrufquén, Río Bueno, Temuco, and Valdivia. Berberine content (Figure 4A) exhibited the highest variability in Temuco and Valdivia, with extreme outliers reaching up to 26,482.20 µg/g in Temuco (C3P26, O), 23,842.81 µg/g in another Temuco accession (C1P24, O), and 20,827.74 µg/g (C2P18, O). Other significant outliers in Temuco included 15,608.98 µg/g (C1P20, O), 10,072.80 µg/g (C1P25, *), and 7359.91 µg/g (C1P21, *). Río Bueno also presented high values, with 15,909.80 µg/g (C2P13, O). Pitrufquén displayed lower median concentrations, but individual accessions reached 9627.04 µg/g (C1P2, *). Nueva Imperial had a notable value of 13,447.86 µg/g (C2P10, O), while Valdivia showed the lowest values, with no high outliers recorded. Palmatine content (Figure 4B) was lower than that of berberine, with the highest value observed in Temuco at 9978.27 µg/g (C2P18, O), followed by 829.21 µg/g (C1P25, O). Río Bueno also presented an elevated value of 674.80 µg/g (C2P13, O) and 440.94 µg/g (C1P7, *), while Pitrufquén reached 1257.91 µg/g (C1P13, O) and 491.92 µg/g (C1P2, *). Nueva Imperial and Valdivia remained consistently low, with maxima of 279.53 µg/g (C2P10, O) and 807.56 µg/g (C1P12, *), and a minimum of 218.42 µg/g (C1P4, *) and 681.34 µg/g (C1P10, *), respectively. The combined berberine + palmatine content (Figure 4C) followed a distribution similar to that of berberine due to its dominance. The highest values were found in Temuco, with peaks at 30,806.02 µg/g (C2P18, O), 26,768.01 µg/g (C3P26, O), 24,644.22 µg/g (C1P24, O), 15,838.52 µg/g (C1P20, O), and 10,902.01 µg/g (C1P25, *). Río Bueno also presented a high value of 16,584.61 µg/g (C2P13, O), while Nueva Imperial reached 13,727.39 µg/g (C2P10, O). Pitrufquén remained moderate at 10,118.96 µg/g (C1P2, *).

**Figure 5:**
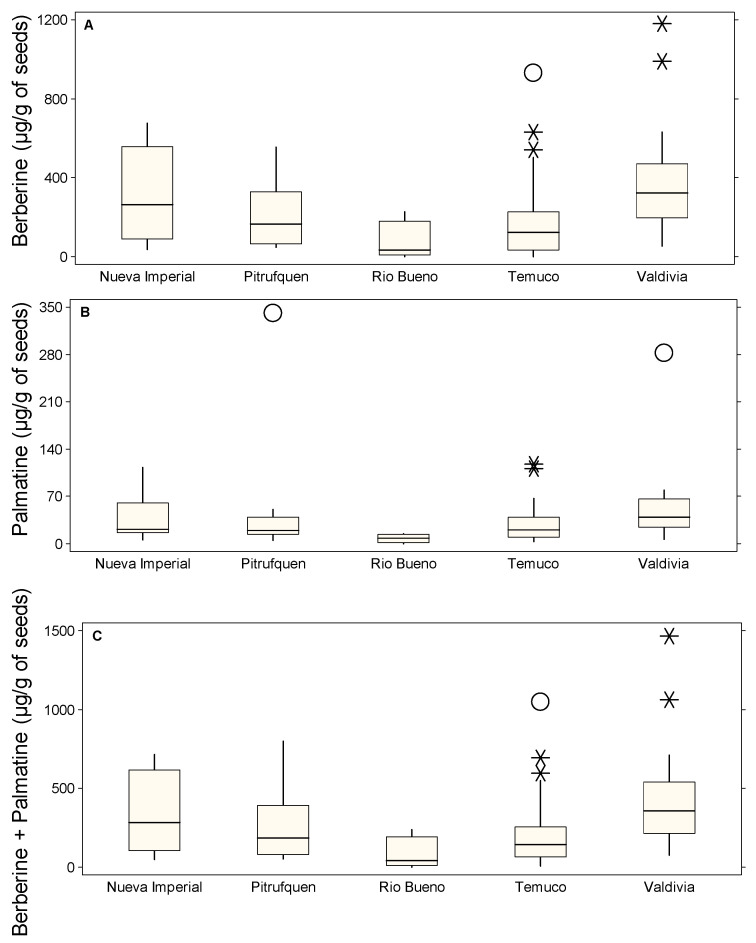
Box plots showing the concentrations of seeds alkaloids (µg/g of DM) in different locations: Nueva Imperial, Pitrufquén, Río Bueno, Temuco, and Valdivia. (**A**) Berberine, (**B**) palmatine, and (**C**) berberine + palmatine. The boxes represent the interquartile range (IQR), the horizontal line within each box indicates the median, whiskers extend to 1.5 times the IQR, and circles denote probable outliers and asterisks denote possible outliers.

Figure 5 represents the distribution of alkaloid content in Michay seeds collected from different localities: Nueva Imperial, Pitrufquén, Río Bueno, Temuco, and Valdivia. In Figure 5A, regarding berberine content, the highest median values were observed in Temuco and Valdivia, with Valdivia also showing the most extreme outliers, such as 1181.75 µg/g (C1P12, *) and 988.68 µg/g (C4P15, *). Temuco presented high variability, with notable values including 930.76 µg/g (C1P22, O), 629.94 µg/g (C1P21, *), and 542.13 µg/g (C2P21, *). Figure 5B shows the palmatine concentrations remained considerably lower than those of berberine across all localities. The highest recorded values were in Pitrufquén, with 340.89 µg/g (C1P2, O), followed by Valdivia, where C1P12 (O) reached 281.93 µg/g. Temuco also presented notable values, with C1P22 () containing 118.11 µg/g and C1P19 reaching 111.32 µg/g. Regarding the total alkaloid content (berberine + palmatine, Figure 5C), since berberine is the dominant alkaloid, the combined content followed a similar distribution. The highest total values were found in Valdivia, where C1P12 (*) accumulated 1463.68 µg/g and C4P15 () reached 1060.18 µg/g. In Temuco, C1P22 (O) reached 1048.87 µg/g, C1P21 presented 694.32 µg/g, while C2P21 had 596.73 µg/g.

**Figure 6:**
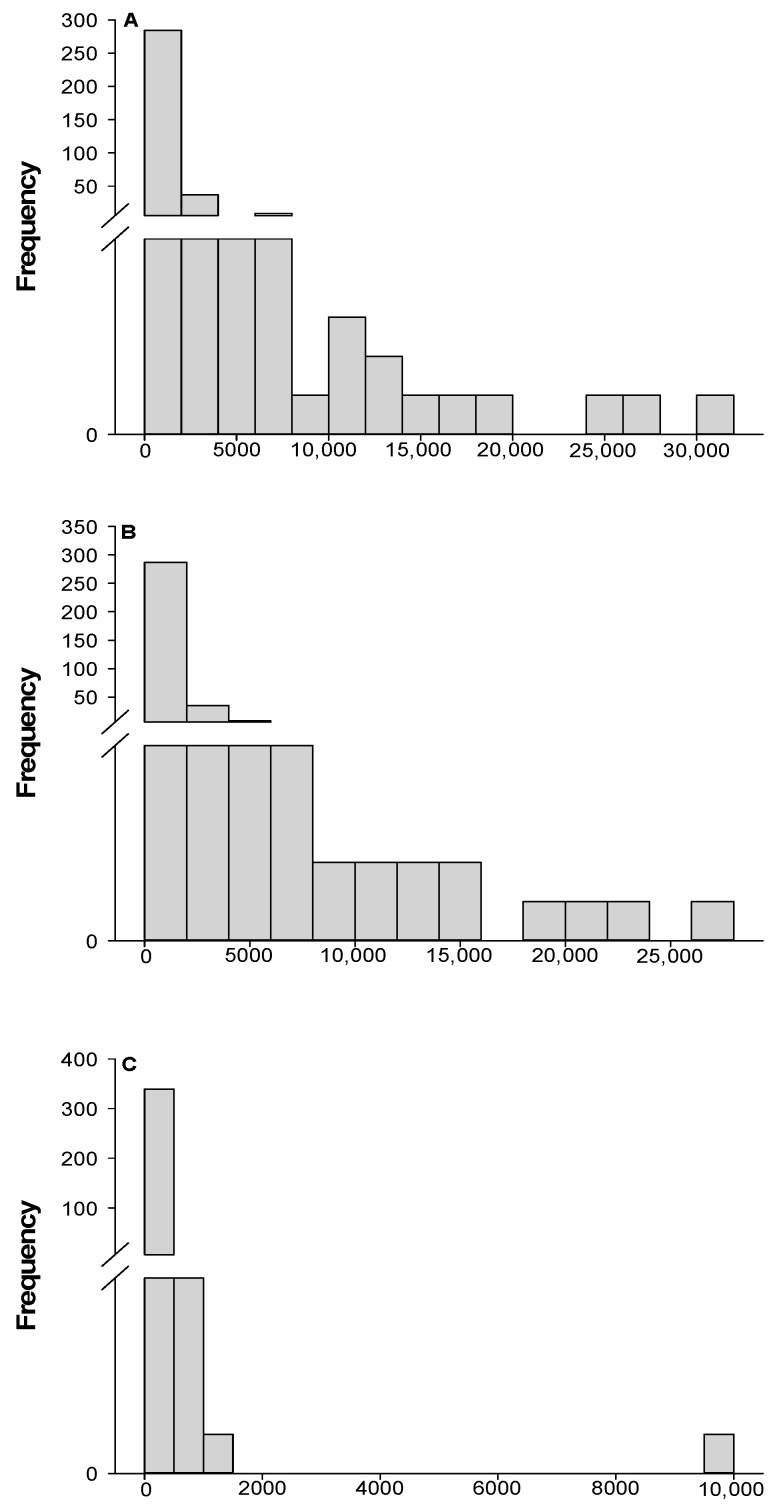
Frequency distribution of alkaloids in Michay accessions: Berberine + palmatine (**A**), berberine (**B**), and palmatine (**C**).

Figure 6A illustrates the total content of berberine and palmatine across all Michay accessions, revealing that C2P18 was the only accession exceeding 30,000 µg/g. This accession stood out with a remarkable combined total of 30,806.02 µg/g, composed of 20,827.74 µg/g of berberine and 9978.27 µg/g of palmatine. The concentration of both alkaloids in this particular accession was significantly higher than in all other accessions, marking it as an extreme outlier among the studied samples. Figure 6B focuses solely on berberine content and highlights C3P26 as the most distinctive accession for this alkaloid, with a striking concentration of 26,482.20 µg/g. This value was considerably higher than the concentrations found in all other accessions, placing it far beyond the range of the remaining samples. Lastly, Figure 6C presents palmatine concentrations, where C2P18 again exhibited the highest value, registering 9978.27 µg/g. This palmatine concentration significantly deviated from the average range observed in other accessions, reinforcing C2P18’s exceptional profile compared to the other samples.

## 3. Discussion

This study has allowed the identification and comparison of the concentrations of berberine and palmatine in different organs of *Berberis darwinii* from various geographic locations, revealing important distribution patterns based on the region of origin. The results suggest that the accumulation of these alkaloid molecules varies significantly between locations, with higher concentrations of both compounds found in the roots of the plants. This follows a trend similar to the findings of Cromwell [35], who reported berberine concentrations of 12,700 µg/g in the main roots and 2900 µg/g in the main stems of 3-year-old wild *B. darwinii* plants. However, it is important to note that Cromwell’s study involved wild *B. darwinii* specimens collected from locations different from those examined in the present study. In 5-year-old plants, the concentration increased to 14,400 µg/g in the main roots and 4000 µg/g in the main stems. For 7-year-old plants, the main roots contained 18,400 µg/g and the main stems 4800 µg/g. In 30-year-old plants, the main roots had a concentration of 28,000 µg/g while the main stems ranged between 26,700 and 49,300 µg/g [35,36].

The differences in berberine and palmatine concentrations across various locations in Chile suggest significant variability in the accumulation of these bioactive alkaloids in *B. darwinii.* Comparisons between plants from each location revealed consistent spatial distribution patterns, with roots accumulating more alkaloids than stems, leaves, and seeds in most cases. Regarding berberine, Temuco and Valdivia stood out with the highest concentrations, whereas for palmatine, the stems exhibited the highest accumulation, particularly in Temuco and Pitrufquén. Additionally, plants from Temuco and Valdivia were found to have the highest concentrations of berberine and palmatine, which could indicate the influence of local environmental factors on the synthesis of these compounds [37]. These findings highlight the potential for selecting *B. darwinii* accessions with greater alkaloid production capacity, which has relevant applications in the pharmaceutical industry.

All these areas exhibit varying climatic and soil characteristics that may influence the alkaloid levels in Michay. For instance, Temuco and Pitrufquén are located in the temperate zone, with relatively high precipitation and cooler temperatures. These regions are characterized by volcanic soils, rich in nutrients and minerals, which are favorable for plant growth. The fertile soils and consistent rainfall in these areas may promote the production of secondary metabolites like alkaloids. Valdivia, with its mild climate and significant rainfall, is home to andic soils rich in organic matter, further supporting plant growth and potentially enhancing alkaloid concentrations. In contrast, Río Bueno and Nueva Imperial, while also in the temperate zone, experience slightly drier conditions. The soils here are sandier and less fertile, with lower organic content and water retention, which may limit the plant’s ability to produce high concentrations of secondary metabolites such as berberine and palmatine.

Moreover, the outlier accessions, particularly C3P26 and C2P18, display exceptional concentrations of berberine and palmatine, respectively. These findings suggest that certain *B. darwinii* accessions may have a higher intrinsic ability to produce these bioactive compounds, potentially due to a combination of favorable environmental conditions and specific genetic traits. The outstanding profile of C2P18, with a combined total of 30,806.02 µg/g of berberine and palmatine, exemplifies the potential of this accession for future breeding programs aimed at enhancing alkaloid production. The high concentration of berberine in accession C3P26 further emphasizes the potential of selecting ecotypes with specialized alkaloid profiles. Previous research has documented that plant populations with unique genetic backgrounds or exposure to particular environmental stressors can produce higher amounts of certain alkaloids [38].

Berberine has been extensively studied for its pharmacological properties, including its antimicrobial, anti-inflammatory, and anticancer effects [39,40,41,42]. Furthermore, its potential lies in the fact that it is generally considered to have low toxicity and minimal side effects [39]. Studies in mice have shown that the LD50 varies significantly depending on the route of administration, with intravenous injection having an LD50 of 9.0 g/kg, intraperitoneal injection at 57.6 g/kg, and no reported LD50 for intragastric administration [40]. In humans, clinical trials assessing the safety of berberine have primarily reported mild gastrointestinal disturbances, such as diarrhea and constipation [36,38,39]. The identification of accessions like C2P18 and C3P26, which contain exceptionally high levels of berberine, offers exciting prospects for pharmaceutical applications. Such ecotypes could be prioritized in breeding programs focused on developing *B. darwinii* cultivars with optimized alkaloid content for therapeutic use.

Moreover, this spatial analysis in Michay stands out because it is the first time that the fruit has been analyzed, finding results never before reported, such as the alkaloid content in the seed and pulp. While it is true that the pulp did not show detectable amounts of berberine or palmatine, the seed did present concentrations, which is potentially interesting. By selecting plants with an interesting content of these alkaloids, it could be possible to generate a selection of “super berries” that not only provide the antioxidants inherent in the fruit but also deliver, through their seeds, quantities of these alkaloids with all their health benefits. Therefore, the generation of functional superfoods through the selection of this plant will open new possibilities for the agricultural food industry, as well as for the nutraceutical and pharmaceutical sectors.

In addition to the factors mentioned earlier, several environmental and genetic factors have been reported to influence alkaloid biosynthesis in plants, and these may help explain the variability observed among *B. darwinii* accessions in this study [43,44]. Environmental variables such as soil nutrient availability, light intensity, temperature fluctuations, and water stress are known to modulate secondary metabolite pathways, including the biosynthesis of isoquinoline alkaloids like berberine and palmatine [18,45]. Additionally, the genetic background of each accession likely plays a critical role, as variations in gene expression related to key biosynthetic enzymes can lead to significant differences in alkaloid accumulation [46]. The observed outliers with exceptionally high concentrations may therefore reflect not only favorable environmental conditions but also superior genetic potential for alkaloid production. Further studies combining transcriptomic and environmental analyses would help clarify the relative contributions of these factors.

While this study provides valuable insights into the alkaloid content of Michay across various localities and plant tissues, a significant limitation is that the measurements of berberine and palmatine content were obtained from a single year of sampling. Given that plant metabolite levels can fluctuate annually due to environmental variability, a one-year study may not fully capture the potential interannual variability in alkaloid production. To address this limitation, future research will aim to conduct multi-year studies to assess the stability and variation of alkaloid concentrations over time. Long-term data will be crucial for determining whether the trends observed in this study hold consistently or if they fluctuate in response to changing climatic conditions, soil health, or other ecological factors. Additionally, while the study includes diverse localities with varying environmental conditions, extending the temporal scope would enable a more comprehensive evaluation of the influence of seasonal variation and long-term climatic shifts on alkaloid synthesis. By incorporating multi-year data, future studies will provide a more robust understanding of the factors influencing alkaloid production in Michay, improving the reliability and applicability of these findings for agricultural and ecological practices in the region.

Additionally, our future plans involve selecting the most promising accessions based on alkaloid content, as well as other desirable traits, such as disease resistance and growth characteristics. These accessions will be clonally propagated to establish more homogeneous populations, which will allow for further research on the inheritance of alkaloid content and the consistency of higher concentrations of berberine and palmatine across multiple years. By expanding the study to include multi-year data, we aim not only to verify the stability of alkaloid concentrations over time but also to better understand the role of specific traits in influencing alkaloid production. This will provide valuable insights into the potential for agricultural and medicinal applications of Michay. Furthermore, selecting stable, high-producing genotypes will help guide future breeding programs and support sustainable cultivation practices.

This study lays the groundwork for the selection and propagation of *B. darwinii* plants with elevated berberine and palmatine content. By analyzing the alkaloid distribution across various plant organs, the findings will offer crucial insights into the variability of these compounds and their potential for medicinal applications. Future research should focus on the long-term stability of alkaloid production in selected high-yielding plants and the optimization of cultivation techniques to further enhance their concentration. This will contribute to the establishment of a sustainable, standardized production system, ensuring a reliable supply of these valuable compounds for pharmaceutical, nutraceutical, and food industries.

## 4. Materials and Methods

**Figure 7:**
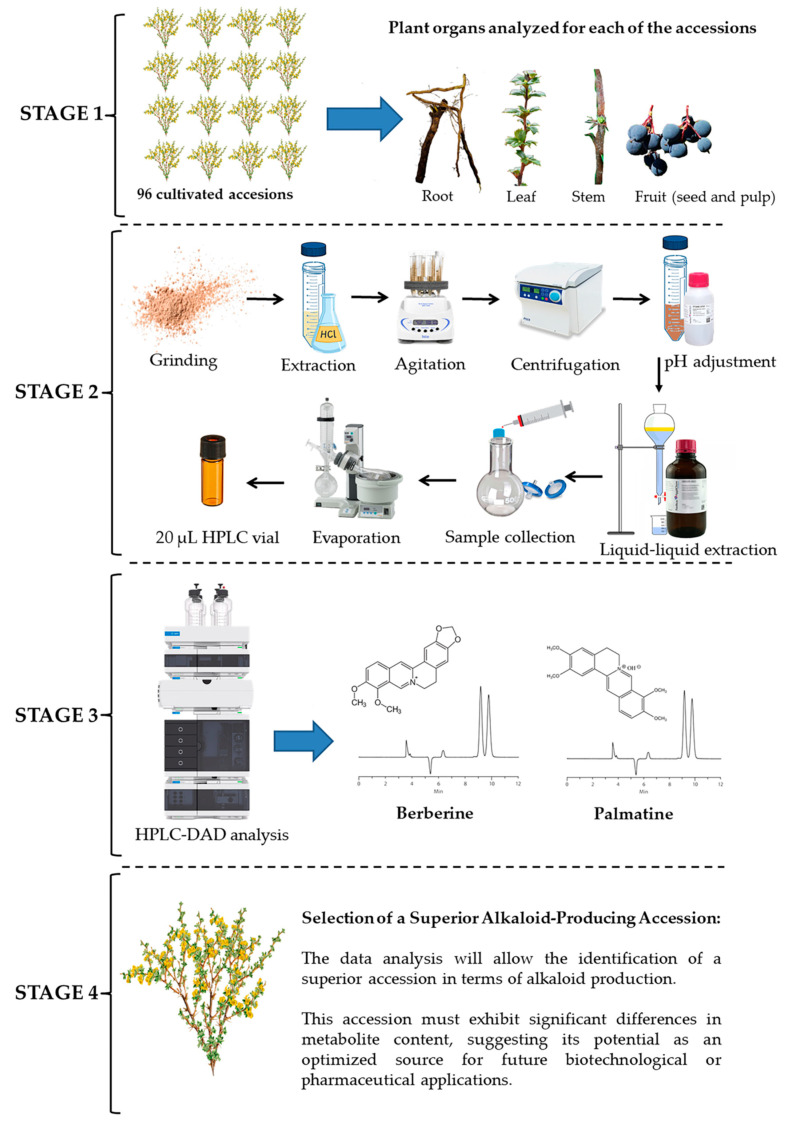
Workflow for selecting superior alkaloid-producing Michay accessions based on berberine and palmatine production from 96 accessions.

A selection process was carried out to identify a superior accession from 96 cultivated accessions based on alkaloid metabolite analysis. Initially, different plant organs (root, leaf, stem, and fruit) were analyzed to determine which parts had the highest alkaloid content. The selected samples underwent grinding, solvent extraction, agitation, centrifugation, pH adjustment, and liquid–liquid extraction to isolate the target compounds. The extracts were then concentrated and prepared in 20 μL vials for chromatographic analysis. HPLC was used to quantify and characterize key alkaloids, such as berberine and palmatine, allowing for metabolic profile comparisons among the ecotypes. Based on these results, a superior accession was identified, displaying significant metabolic differences, suggesting its potential as an optimized source for biotechnological or pharmaceutical applications (Figure 7).

### 4.1. Establishment of Plant Material and Experimental Design

In June 2019, a garden experiment was conducted at the Huichahue Experimental Field, belonging to the Agro-Aquaculture Nutritional Genomics Center (CGNA) (Padre Las Casas commune, coordinates 38°50′16.34″ S; 72°26′53.82″ O). Wild Michay plants were collected from their native habitats in various locations within the La Araucanía Region, including Temuco (38°42′50.34″ S; 72°35′49.04″ O) (34 plants), Pitrufquén (38°59′30.03″ S; 72°36′50.51″ O) (13 plants), and Nueva Imperial (38°44′47.70″ S; 72°58′47.55″ O) (17 plants), as well as from the areas of Río Bueno (40°19′36.23″ S; 72°56′44.91″ O) (14 plants) and Valdivia (18 plants) in the Los Ríos Region. The Michay orchard was then established at the Huichahue experimental field with a north–south orientation. Four 40 cm long by 50 cm wide ridges were created, with a distance of 2.6 m between rows and 0.9 m between plants within rows, allowing 24 plants to be placed per ridge, resulting in a total of 96 different accessions with a planting density of 4274 plants/ha. (Each row was originally planted with 26 plants, but some failed to establish, resulting in a final count of 24 plants per row. To standardize the identification of each established accession, the following nomenclature was assigned: C1P1–P26 for row 1, where “C1” represents the row and “P1–P26” denotes the established plants. The same system applies to C2P1–P26 for row 2, C3P1–P26 for row 3, and C4P1–P26 for row 4. This approach ensures clear and organized plant identification, facilitating monitoring and data collection.) The experimental design involved grouping the Michay plants into sets of three based on the five collection zones mentioned and randomly placing these groups on the ridges.

The entire orchard was enclosed with an open mesh for protection. Additionally, a black weed-control mesh was placed on each of the ridges to limit weed presence. The soil in the orchard is classified as the Temuco series (Andosols), consisting of deep soils formed by very ancient volcanic ash found in the central plains, located at an altitude of 100 to 300 m above sea level, with a loamy clay texture and dark brown color. Soil analysis revealed the following values: N = 16 mg/kg, P = 7 mg/kg, K = 137 mg/kg, Mn = 0.64 mg/kg, Zn = 0.21 mg/kg, Cu = 0.52 mg/kg, Fe = 28 mg/kg, B = 0.3 mg/kg, pH = 6.1, and organic matter = 17% (Universidad de la Frontera). Prior to planting, the soil was plowed to a depth of 40 cm, harrowed, and then mounded to a height of 40 cm.

### 4.2. Sample Collection

Samples of leaves, stems (main), roots (subsidiary), and fruits (seeds and pulp) were collected from *B. darwinii* plants. For the collection, plants were carefully selected from different accessions in the experimental orchard. The leaves were harvested from the upper, middle, and lower sections of the plants to represent different developmental stages [47]. Stems were collected from the main trunk and lateral branches [48]. Roots were carefully excavated, ensuring that the samples were free from soil contamination [16]. The fruits were picked when they were fully ripe, and they were carefully divided to separate the seeds from the pulp [49]. All collected samples were stored immediately at −80 °C until their analysis [50].

### 4.3. Alkaloid Extraction Procedure

Leaves, stems, roots, and fruits (seeds and pulp) of *B. darwinii* collected were frozen at −80 °C for 24 h. The root samples were carefully washed with distilled water to prevent soil contamination during the process and were disinfected with a 0.1% sodium hypochlorite solution (Merck KGaA, Darmstadt, Germany) [16]. Then, the samples were lyophilized for 48 h. Afterward, they were ground to obtain 150 mg of fine powder using a grinder. Next, 5 mL of 0.5 N HCl was added to the 150 mg of plant material in a 15 mL Falcon tube, followed by vigorous shaking in a vortex for 48 h in the dark. After shaking, the Falcon tube was centrifuged at 4000 rpm for 15 min, and the supernatant was collected and stored in another Falcon tube. The extraction process was repeated with the resulting pellet, and both extractions were combined. To neutralize the alkaloid extract, the pH was adjusted to 13 by adding 700 µL of 10 N NaOH (Merck KGaA, Darmstadt, Germany). A liquid–liquid extraction was performed by placing the 10 mL of extract into 100 mL separation funnels, to which 10 mL of chloroform (chromatographic grade, Sigma-Aldrich, St. Louis, MO, USA) was added. The funnels were shaken for 2 min to ensure phase stabilization, and then the chloroform was collected into a 250 mL flask. This process was repeated four times. Once the extraction was completed, the solvent was evaporated under vacuum using a rotary evaporator. Finally, 2 mL of 10% ethanol (HPLC grade, Sigma-Aldrich, St. Louis, MO, USA) was added and shaken inside the flask to remove any alkaloid residues from the walls. The resulting samples were filtered through PVDF membranes of 0.22 µm (0.2 µm × 25 mm) and transferred to 2 mL tubes for injection into the HPLC [51,52]. Recovery assays were conducted by spiking known concentrations of berberine and palmatine into the plant matrix prior to extraction. The recovery rates for both alkaloids were 95%, demonstrating the method’s accuracy and its ability to effectively overcome potential interferences from the plant matrix.

### 4.4. HPLC-DAD Analysis

**Figure 8:**
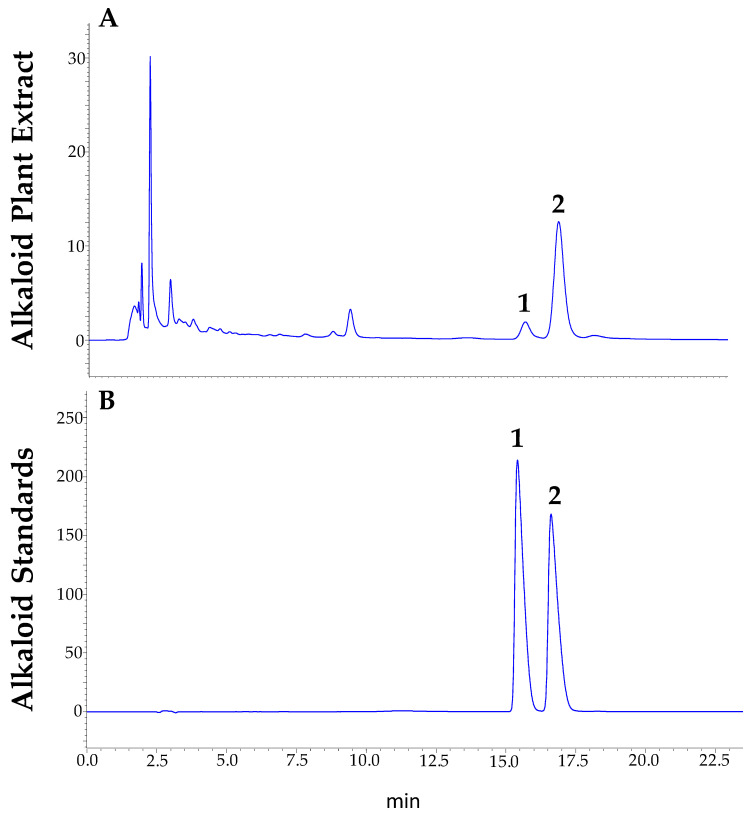
Representative HPLC chromatogram showing the separation of berberine (2) and palmatine (1) under the described analytical conditions: (**A**) represents the chromatogram of a *Berberis darwinii* extract, and (**B**) represents the chromatogram of the standard mixture.

Chromatographic analysis was performed using a Shimadzu LC-2050C 3D high-performance liquid chromatograph, equipped with a Restek ROC C18 column (4.6 × 250 mm × 5 µm). The mobile phase consisted of a mixture of acetonitrile and a solution containing 20 mM KH_2_PO_4_ (pH 2.8) in a 25:75 ratio. The column temperature was maintained at 40 °C, and detection was carried out at a wavelength of 229 nm for berberine and 344 nm for palmatine. The flow rate was set at 1.0 mL/min, and 10 μL of the sample was injected. The elution process was performed in isocratic mode. For the calibration curve, stock solutions of berberine and palmatine were prepared by dissolving each alkaloid in methanol to obtain a concentration of 500 mg/mL. These solutions were then diluted with methanol to generate working standard solutions. Calibration curves were established for both compounds to evaluate the linearity of the method, using standard solutions ranging from 0.0976 to 100 µg/mL. The working range was defined as 0.43–12.5 µg/mL for berberine and 0.14–12.5 µg/mL for palmatine. The identification of berberine and palmatine was based on the comparison of retention times of the peaks with those of pure standards. Both alkaloids showed excellent linearity, with correlation coefficients (R²) of 0.9999, confirming the reliability of the method for quantitative analysis within the selected concentration ranges. To establish the limits of detection (LOD) and quantification (LOQ), serial dilutions of each stock standard solution were prepared using MeOH as the solvent. Each of these solutions was injected into the HPLC instrument until a signal-to-noise (S/N) ratio of 3 was reached for the LOD and a ratio of 10 for the LOQ of each alkaloid compound. Based on this approach, the LOD and LOQ for berberine were determined to be 0.14 µg/mL and 0.43 µg/mL, respectively, while for palmatine they were 0.04 µg/mL and 0.14 µg/mL. These values are method-based and apply across all plant organs analyzed (roots, stems, leaves, and seeds), as the same analytical procedure was used for all matrices. Berberine chloride (purity, >98%), palmatine chloride (purity, >97%), and all analytical grade solvents used for extractions were purchased from Sigma Aldrich (St. Louis, MO, USA) [53]. Berberine and palmatine were successfully separated under these chromatographic conditions, showing sharp and well-resolved peaks with no overlapping, as illustrated in Figure 8, confirming the efficiency of the method.

### 4.5. Statistical Analysis

The statistical software Statistix 10 (Tallahassee, FL, USA) was used for data analysis. To compare the content of berberine, palmatine, and their sum by individual locality, as well as the content of these compounds separately across the different tissues of the 96 Michay accessions, an analysis of variance (ANOVA) (*p* ≤ 0.05) was conducted, followed by Tukey’s test to assess significant differences. Prior to ANOVA, data were subjected to two preliminary tests: the Shapiro–Wilk test to assess the normality of residuals, and Levene’s test to evaluate the homogeneity of variances among groups. Where deviations from normality were detected, a logarithmic transformation was applied to improve distribution. However, at this stage, our primary goal was not to emphasize variance comparison but to characterize the variability within and between groups, particularly to identify outlier accessions that may represent interesting patterns or extremes for further analysis. Furthermore, to detect the outlier accessions, a box plot was created. Each box plot was composed of a box and two whiskers, with the box bisected by a line at the median value. Extreme values are displayed as “*” for possible outliers and “O” for probable outliers. Possible outliers were values outside the box boundaries by more than 1½ times the size of the box, while probable outliers were values outside the box boundaries by more than 3 times the size of the box. Finally, a histogram was constructed for berberine, palmatine, and their sum to analyze the ecotype outliers in the total content of these alkaloids.

## 5. Conclusions

This study has allowed the identification and comparison of the concentrations of berberine and palmatine in different organs of *Berberis darwinii* from various geographic locations, revealing important distribution patterns based on the region of origin. The results suggest that the accumulation of these alkaloids varies significantly between locations, with higher concentrations of both compounds in the roots of the plants. Additionally, plants from Temuco and Valdivia were found to have the highest concentrations of berberine and palmatine, which could indicate the influence of local environmental factors on the synthesis of these compounds. This finding opens the possibility of selecting *B. darwinii* accessions with greater potential for the production of these alkaloids, which has relevant applications in the pharmaceutical industry. In summary, the variability observed between the different locations highlights the importance of considering geography and ecological factors in the study of species with bioactive properties. In conclusion, this study has identified key ecotypes of *B. darwinii* with significant variations in the concentrations of berberine and palmatine across different accessions. Notably, the accession C2P18 exhibited the highest combined total of both alkaloids, with 30,806.02 µg/g, making it an extreme outlier among the samples studied. Additionally, C3P26 was distinguished for its exceptionally high berberine content, and C2P18 again stood out for its remarkable palmatine concentration. These ecotypes demonstrate considerable potential for selection in breeding and propagation programs aimed at achieving a consistent and stable production of these valuable bioactive compounds. The identification of such ecotypes is crucial for improving *B. darwinii* as a reliable source for pharmaceutical and industrial applications.

## Figures and Tables

**Table 1:** Concentration of berberine and palmatine (µg/g dry weight) in different plant organs (leaf, stem, root, and seed) from five collection zones. Values represent the mean, standard deviation (SD), coefficient of variation (CV, %), minimum, and maximum concentrations. The CV was used to assess intra-sample variability across replicates within each location and organ.

Collection Zone		Mean (µg/g)	Standard Deviation	Coefficient of Variation	Minimum	Maximum
Nueva Imperial	Leaf					
	Berberine	102.88	135.56	131.76	0.0001	511.02
	Palmatine	4.79	7.04	147.07	0.0001	24.78
Pitrufquén						
	Berberine	44.54	72.71	163.23	0.0001	233.82
	Palmatine	3.72	5.01	134.72	0.0001	18.71
Rio Bueno						
	Berberine	109.51	153.51	140.19	3.8800	493.10
	Palmatine	9.17	9.80	106.90	1.0200	30.23
Temuco						
	Berberine	82.52	106.89	129.52	0.0001	455.63
	Palmatine	9.98	13.83	138.58	0.0001	62.57
Valdivia						
	Berberine	20.88	25.71	123.17	0.0001	89.36
	Palmatine	2.88	2.83	98.07	0.0001	9.12
Nueva Imperial	Stem					
	Berberine	1059.00	1102.90	104.14	13.800	3619.80
	Palmatine	19.89	40.21	202.10	0.0001	170.03
Pitrufquén						
	Berberine	1540.90	1678.90	108.96	0.0001	5101.30
	Palmatine	55.37	91.65	165.51	0.0001	342.78
Rio Bueno						
	Berberine	2020.10	1740.60	86.16	136.00	6639.60
	Palmatine	51.16	75.47	147.52	0.0001	265.32
Temuco						
	Berberine	1934.60	1761.80	91.06	145.73	6544.20
	Palmatine	100.95	137.21	135.92	0.0001	533.41
Valdivia						
	Berberine	828.20	954.60	115.26	0.0001	3766.30
	Palmatine	34.02	34.52	101.48	0.0001	99.11
Nueva Imperial	Root					
	Berberine	1721.20	3153.50	183.21	0.0001	13,448.00
	Palmatine	50.77	83.11	163.69	0.0001	279.53
Pitrufquén						
	Berberine	2404.00	2604.60	108.34	111.77	9627.00
	Palmatine	186.93	336.03	179.76	0.0001	1257.90
Rio Bueno						
	Berberine	2389.50	4394.30	183.90	0.0001	15,910.00
	Palmatine	105.89	205.85	194.40	0.0001	674.80
Temuco						
	Berberine	3871.00	6995.40	180.71	0.0001	26,482.00
	Palmatine	385.91	1680.60	435.50	0.0001	9978.30
Valdivia						
	Berberine	4262.20	5553.90	130.31	0.0001	18,296.00
	Palmatine	165.04	237.87	144.13	0.0001	807.56
Nueva Imperial	Seed					
	Berberine	313.03	240.17	76.72	35.840	674.99
	Palmatine	39.38	32.74	83.13	5.6300	113.24
Pitrufquén						
	Berberine	214.49	174.50	81.35	46.270	554.25
	Palmatine	54.19	101.65	187.57	4.6500	340.89
Rio Bueno						
	Berberine	81.53	94.74	116.20	0.0001	226.95
	Palmatine	7.84	6.40	81.69	0.0001	14.57
Temuco						
	Berberine	194.11	227.18	117.04	1.2600	930.76
	Palmatine	29.76	30.40	102.17	3.2300	118.11
Valdivia						
	Berberine	409.03	321.31	78.555	52.350	1181.80
	Palmatine	58.68	67.56	115.12	6.8300	281.93

## Data Availability

All data supporting the findings of this study are fully available within the manuscript. No additional datasets were generated or analyzed beyond those presented in the text.
